# The antiphospholipid syndrome may induce non-thrombotic internal jugular vein stenosis: two cases report

**DOI:** 10.1186/s12883-020-02035-1

**Published:** 2021-01-07

**Authors:** Si-ying Song, Gary Rajah, Yu-chuan Ding, Xun-ming Ji, Ran Meng

**Affiliations:** 1 Department of Neurology, Xuanwu Hospital, Capital Medical University, Chang Chun road 45, Xicheng, Beijing, China; 2 Advanced Center of Stroke, Beijing Institute for Brain Disorders, Beijing, 100053 China; 3 Department of China-America Institute of Neuroscience, Xuanwu Hospital, Capital Medical University, Beijing, 100053 China; 4 Department of Neurosurgery, Jacobs School of Medicine and Biomedical Sciences, University at Buffalo, Buffalo, NY USA; 5 Department of Neurosurgery, Wayne State University School of Medicine, Detroit, MI 48201 USA

**Keywords:** Antiphospholipid syndrome, Non-thrombotic internal jugular vein stenosis, Neuroimaging, Case report

## Abstract

**Background:**

Antiphospholipid syndrome (APS) is associated with a high incidence of thrombotic events, either arterial thrombosis or venous thrombosis. However, APS-related non-thrombotic venous stenosis is rarely reported.

**Case presentation:**

This study described two cases of young women with APS-related internal jugular vein stenosis (IJVS) and reviewed current literature on this issue, including clinical features, diagnosis, and treatment.

**Conclusions:**

IJVS is a rather rare complication of APS. Two cases were reported for the first time that high titer of antiphospholipid antibodies (aPL) might mediate direct vessel wall damage and further induce venous stenosis despite long-term standardized anticoagulation to prevent thrombus formation. Therefore, dynamic monitoring of autoantibodies and concomitant use of anticoagulants and corticosteroids may be necessary to the management of APS and its complications.

**Supplementary Information:**

The online version contains supplementary material available at 10.1186/s12883-020-02035-1.

## Background

Antiphospholipid syndrome (APS) is characterized by venous or arterial thrombosis in young adults with persistent laboratory evidence of high titer of antiphospholipid antibodies (aPL) [1–3]. However, APS-induced cerebral venous damage may present various entities, including thrombotic or non-thrombotic venous stenosis. Non-thrombotic stenosis is frequently caused by attacks from autoimmune antibodies, with a pathological characteristic of vasculitis and without common vascular risk factors, such as hypertension, diabetes mellitus, hyperlipidemia, or smoking. To our knowledge, APS-related-non-thrombotic venous stenosis is rather rare and never reported before due to the misclassification or ambiguous classification of APS thrombotic complications when patients are combined with other multiple vascular risk factors in clinical settings. Herein, we reported two young female patients of APS with non-thrombotic internal jugular vein stenosis (IJVS) presenting with intracranial hypertension symptoms even after long-term standardized anticoagulation and corticosteroids. We presumed that APS might trigger autoimmune inflammatory damage to venous walls, then leading to edema and thickening of the walls, eventually resulting in non-thrombotic IJVS. To our knowledge, this is the first report up to now. We believed this report could provide an essential reference for physicians.

## Case presentation

### Scientific justification of crucial concept

APS is an autoimmune disease, in which aPL (anticardiolipin antibodies and lupus anticoagulants) react against proteins that bind to anionic phospholipids on plasma membranes. Non-thrombotic stenosis is defined as vasculitic lesions from other vasculopathies (e.g. APS, Sjögrens syndrome, and Behçet’s disease), or compression of vessel walls by surrounding bones/vessels/lymph nodes, instead of vessel wall lesions from atherosclerotic plaques, termed as thrombotic stenosis, or reversible cerebral vasoconstriction syndrome**.**

### Case introduction

Two young female patients of APS were confirmed as IJVS by Digital subtraction angiography (DSA) and Magnetic resonance black blood thrombus imaging (MRBTI). Both of them did not show any vascular risk factors, which further indicated the classification of the venous stenosis was non-thrombotic and primarily caused by attacks from aPL. Although these two cases had similar demographic information, the clinical development of the primary disease and related complications varied due to whether or not receiving standardized therapy of corticosteroid.

### Case 1

A 33-year-old woman complained of a five-year worsening blurry vision, concomitant with an intermittent headache on temporal regions, and nocturnal bilateral tinnitus. She was found progressive memory loss for the last six months, without nausea and vomiting, weakness or sensory symptoms in the face and limbs, sphincter incontinence, loss of consciousness, and seizures. Comorbid medical issues included a 5-year history of APS (confirmed primary APS at Beijing Union Hospital) and deep vein thrombosis (DVT) in the left leg. Thereafter, she underwent a therapy of corticosteroid for 1-year plus standardized anticoagulation till this admission. She denied a family history of thrombotic diseases. On physical examination, her body temperature was 37.0 °C, blood pressure was 128/78 mmHg, heart rate was 79 beats/minute, and respiratory rate was 20 breath/minute. Her Body mass index (BMI) was 31.1. No focal neurological signs were found.

Both the platelet count (373*10^9/L) and plateletcrit (0.36%) were elevated in her baseline peripheral blood test. The level of protein C exceeded the normal upper limit (155%) slightly. Other items related to coagulation showed no positive significance. All these results of serological tests, including aPL, antinuclear antibodies (ANA), antineutrophil cytoplasmic antibodies (ANCA), and complements C3 and C4 were negative. The lumbar puncture opening pressure (LPOP) was 250 mmH_2_O with a normal cerebrospinal fluid profile. Funduscopic examination revealed bilateral optic disc edema (Frisén score = 2).

**Fig. 1:**
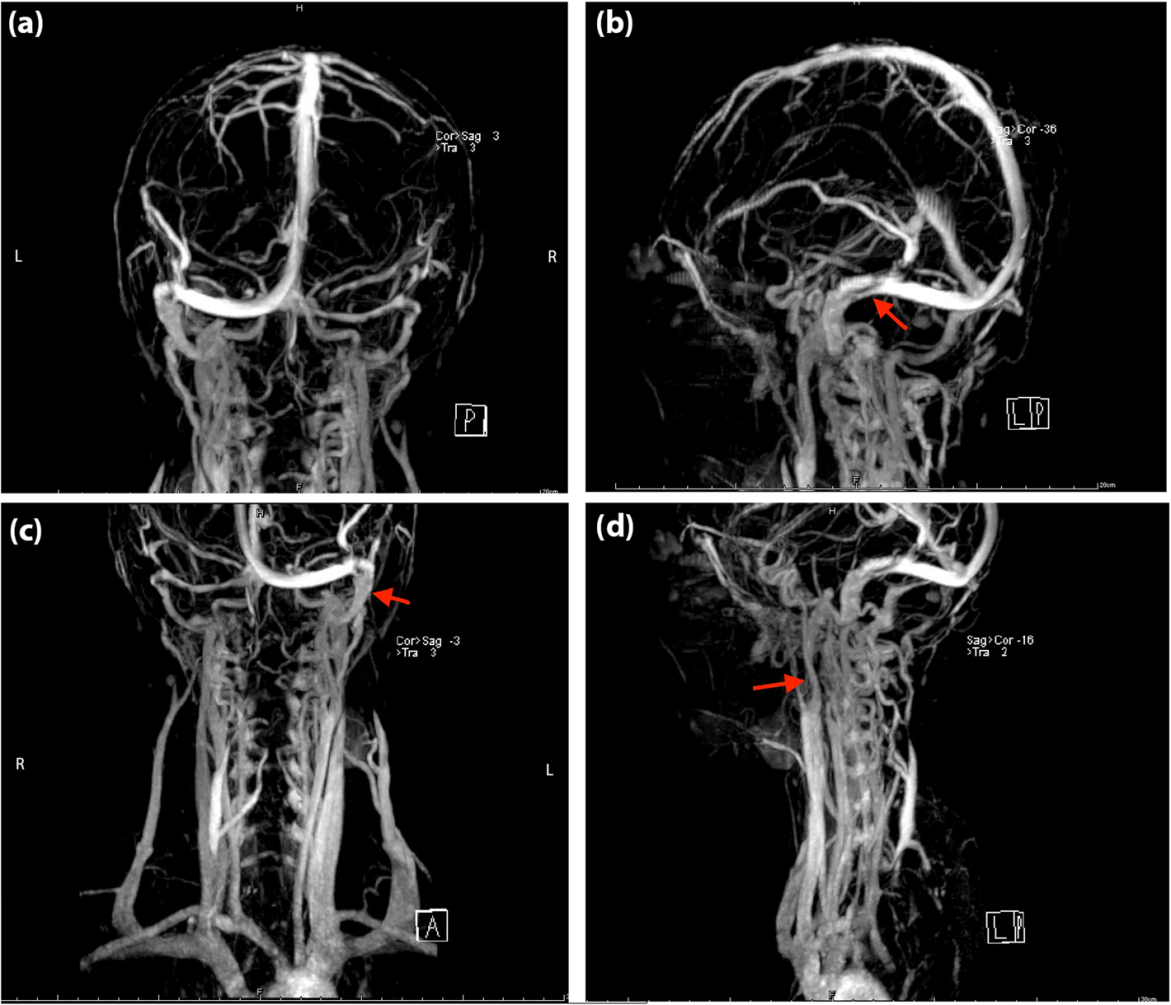
Magnetic resonance venography images of the head (**a**, **b**) and neck (**c**, **d**) in Case 1. The red arrow indicates the focal stenosis of the internal jugular vein and cerebral vein sinus

**Fig. 2:**
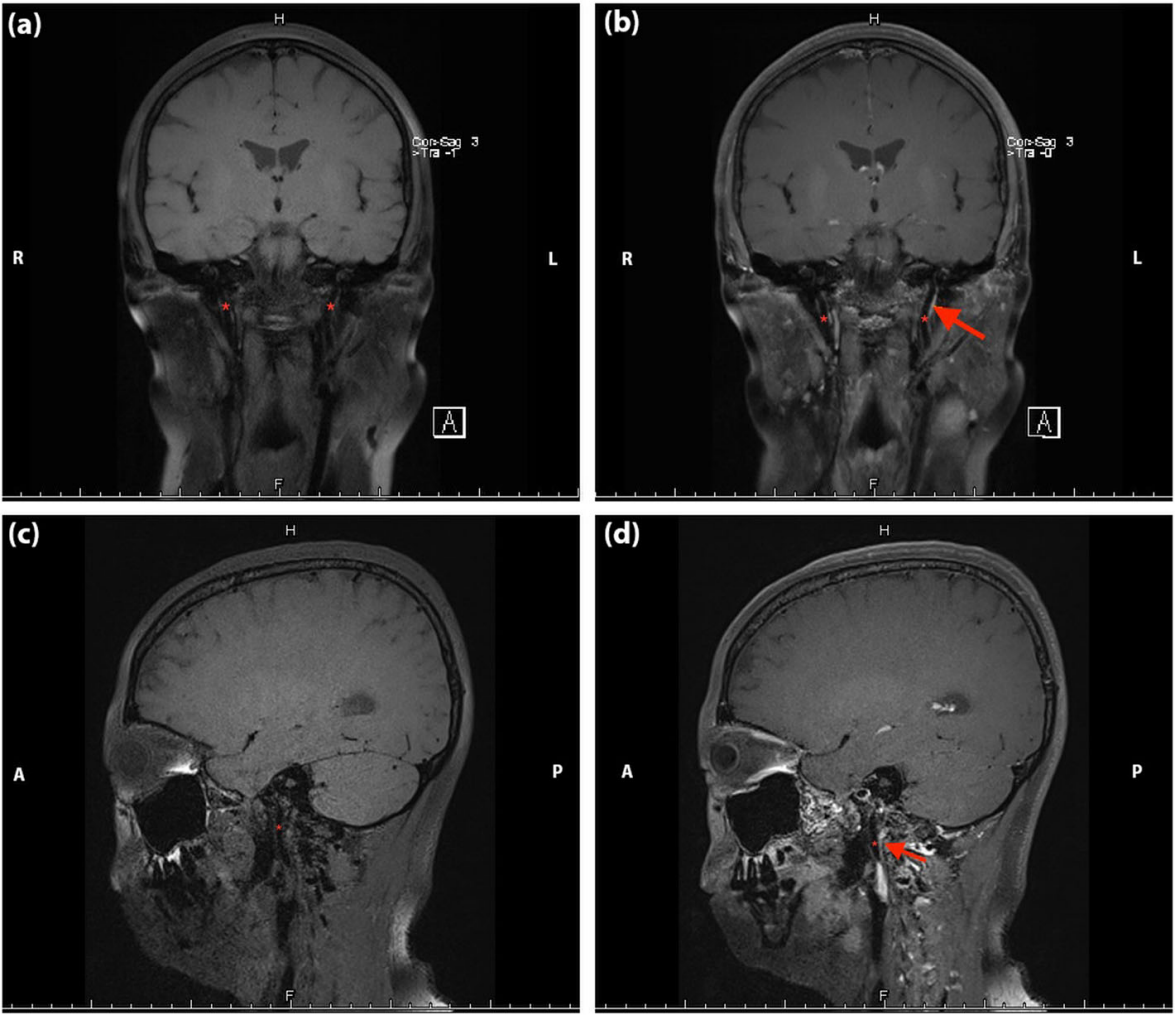
Non-contrast enhanced (**a**, **c**) and contrast-enhanced (**b**, **d**) magnetic resonance black blood thrombus imaging of head in Case 1. The red asterisk represents internal jugular veins, and the red arrow indicates the focal stenosis

Color Doppler ultrasound displayed venous valve insufficiency involving bilateral common femoral veins, right deep femoral vein, and left superficial femoral vein. Both Transcranial Doppler (TCD) and carotid ultrasound showed no abnormalities. However, jugular vein ultrasound revealed a small diameter in the upper segment (J3) of the right IJV with decreased flow volume (Supplementary Table 1). Contrast-enhanced magnetic resonance venography (CE-MRV) of the brain and neck identified the stenosis located at left transverse sigmoid sinus junction (Fig. 1: a, b) and the upper segment (J3) of the left IJV (Fig. 1: c, d). No thrombus was found in either cerebral venous sinus or IJV in the MRBTI (Fig. 2). DSA further confirmed severe non-thrombotic stenosis in the left IJV. All the aforementioned symptoms and signs attenuated after undergoing two-week of corticosteroid (25 mg/day of oral prednisone) treatment. Anticoagulation was then continued (20 mg of oral Rivaroxaban daily).

### Case 2

A 32-year-old woman presented with two-week progressively aggravated pains in her head and neck with nausea and vomiting. Double vision lasted for three days after she was recovered from pneumonitis. She once had the similar symptoms two years ago, with LPOP of 330 mmH_2_O measured at a local hospital. She was diagnosed as idiopathic intracranial hypertension (ICH) and given mannitol intravenously. Her intracranial pressure (ICP) was decreased to 150 mmH_2_O before discharge. However, two weeks prior to this admission, her symptoms reappeared, with LPOP of over 400 mmH_2_O. Intravenous mannitol injection could not relieve her symptoms this time.

On this admission, she complained of severe left-side headache in a spasmodic manner, accompanied by persistent nausea and blurry visions, as well as occasionally vomiting undigested food. Ophthalmologic examination revealed papilledema surrounding with small local bleeding spots (Frisén score = 4). Her vital signs were as follows: body temperature, 36.5 °C; blood pressure, 115/68 mmHg; heart rate, 78 beats/minute; and respiratory rate, 20 breath/minute. Her BMI was 20.1. Neurological examination revealed mild left abduces palsy.

**Fig. 3:**
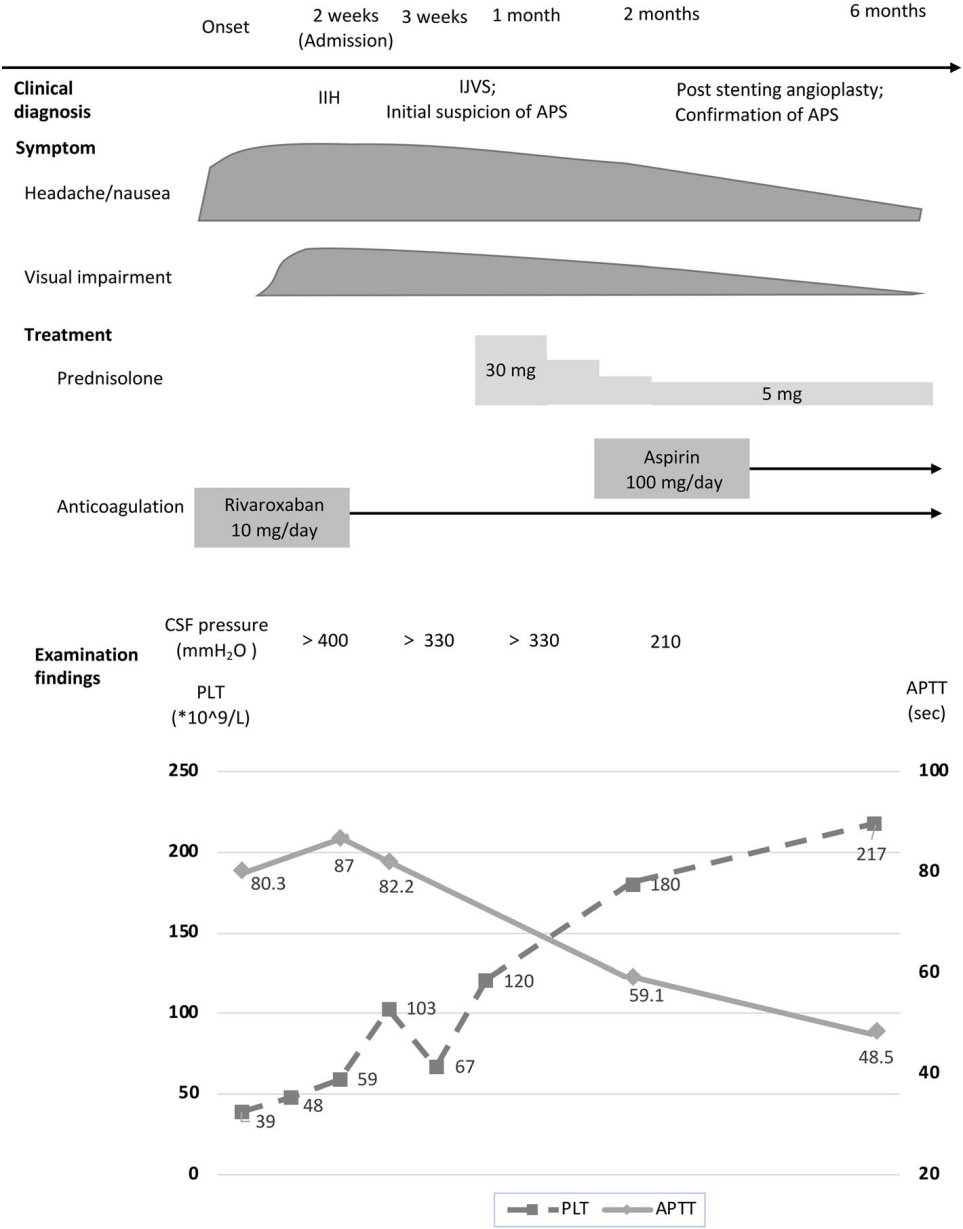
Dynamic changes of laboratory results and clinical process of the Case 2

**Fig. 4:**
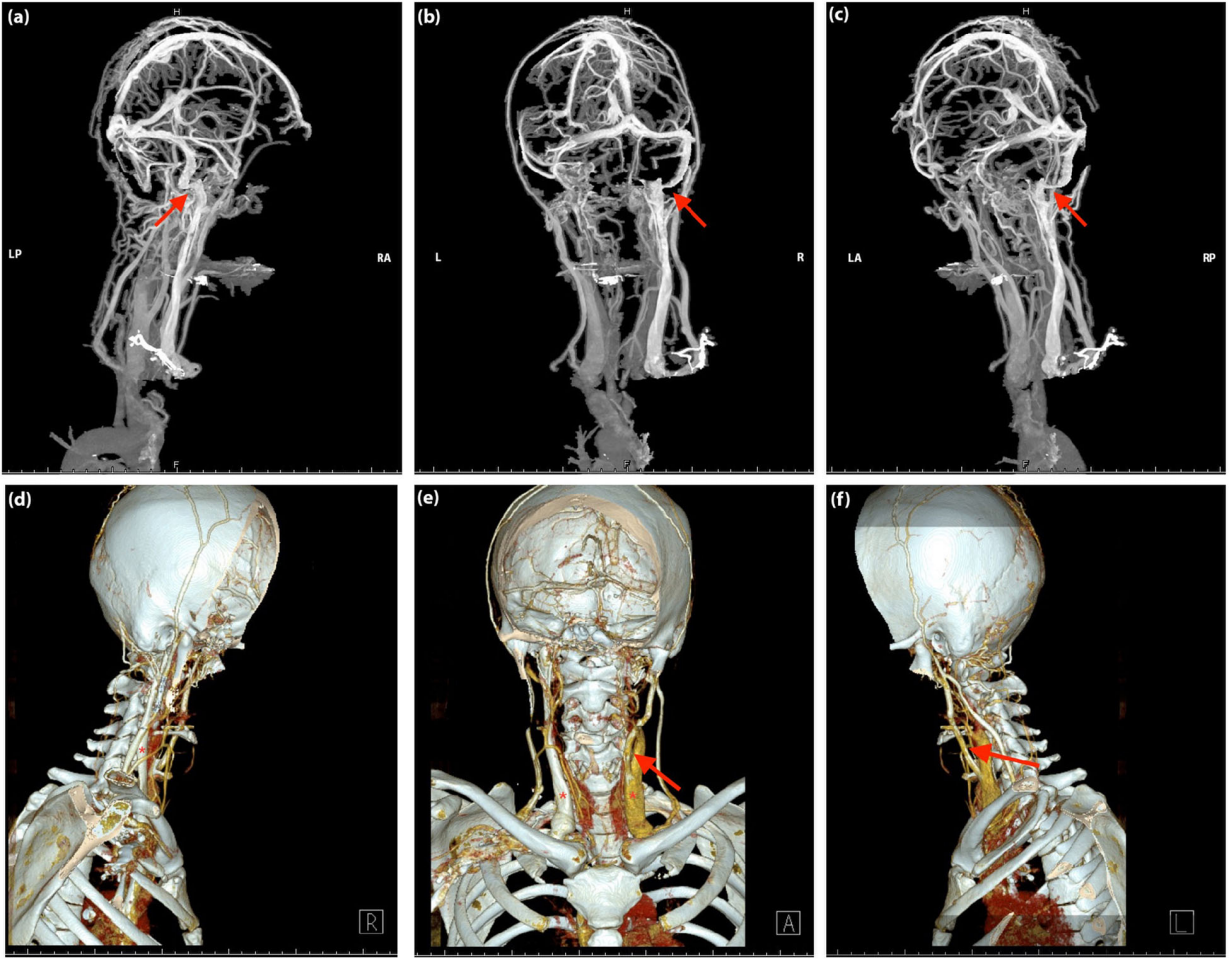
3D-computed tomography venography images (CTV) (**a**-**c**) and 3D-CTV (**d**-**f**) with bone remodeling of the head and neck in Case 2. 3D-CTV with the bone remodeling excluded possibility of IJVS related to bone compression

**Fig. 5:**
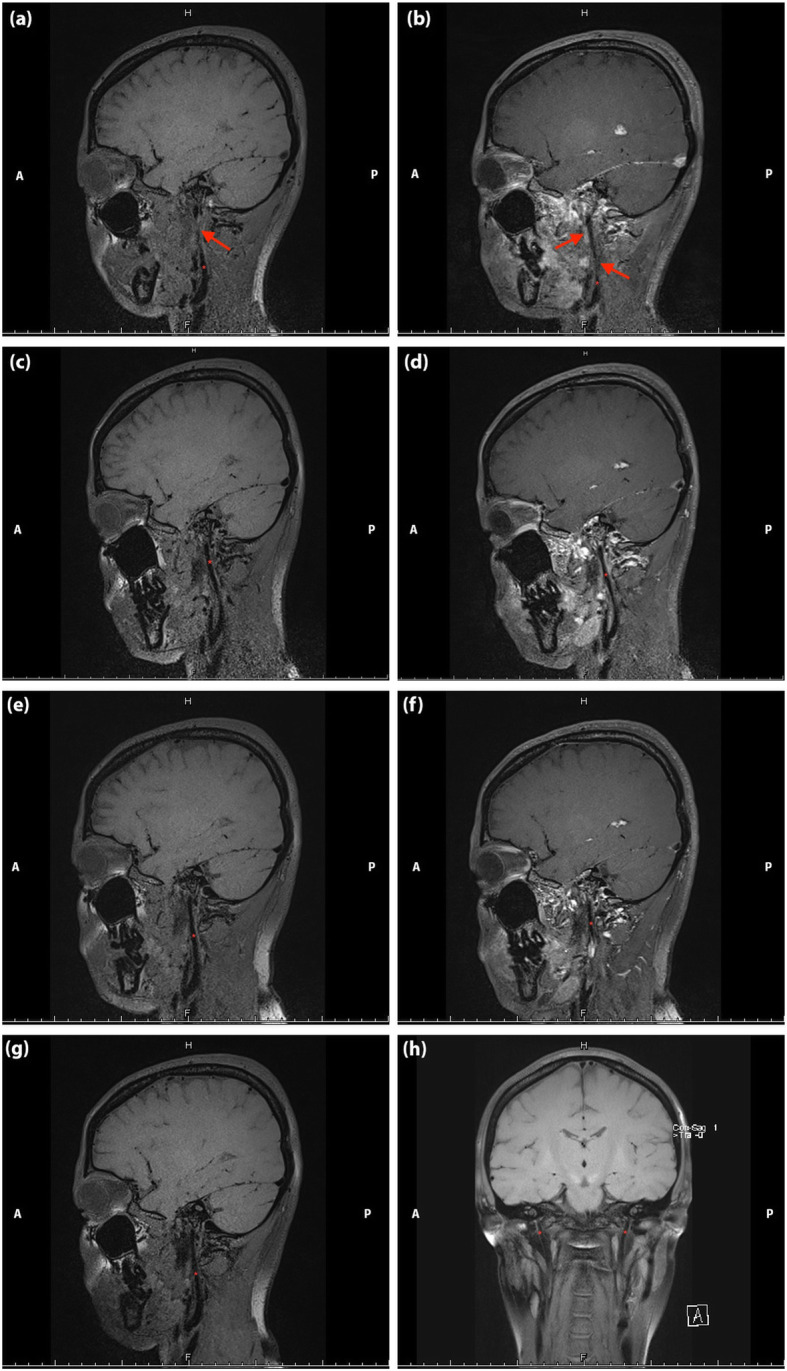
Non-contrast enhanced (a, c, e, g, h) and contrast-enhanced (b, d, f) magnetic resonance black blood thrombus imaging of head in Case 2. **a** and **b**: on admission; **c** and **d**: post-stenting at one week; **e** and **f**: post-stenting at one month; **g** and **h**: post-stenting at six-month. The stenosis attenuated gradually (the red asterisk represents internal jugular vein and the red arrow indicates the focal stenosis)

Peripheral blood and cerebral spinal fluid examination results were summarized in Supplementary Table 2. Dynamic changes of abnormal results were shown in Fig. 3. Platelet count decreased dramatically to 39*10^9/L on the first day and was gradually back to normal range in one week. The activated partial thromboplastin time (APTT) was 80.3 s (normal range: 25–43.5 s). The value of protein S was lower than the normal lower limit (35%). The level of serum IgG increased (16.7 g/L), while the levels of serum complements C3 and C4 were below lower reference level (C3: 0.79–1.52; C4: 0.16–0.38). Anti-β2-glycoprotein 1 (anti-β2GPI) antibody level (50.0 RU/mL) was more than two times than the upper limit (normal range: 0.0–20.0 RU/mL). ANA and ANCA were negative. In terms of neuroimaging, we confirmed the left IJVS and excluded both arterial and venous thrombosis via computed tomography venography (Fig. 4) and MRBTI (Fig. 5: a, b). She was prescribed with oral Rivaroxaban 10 mg/day after admission. A lumbar puncture followed up after the platelet count turned to normal showed that ICP was more than 330 mmH_2_O (three weeks after onset). Finally, local stenosis at J3-segment of left IJV was confirmed by DSA and corrected by endovascular stenting (Fig. 4: c, d). The headache and visual disorder were mitigated immediately after stenting, whereas LPOP was still more than 330 mmH_2_O. She then underwent Rivaroxaban 10 mg/day combined with prednisone 30 mg/day. The dose of prednisone was gradually reduced to 5 mg/day afterward.

About one month after admission (two months after onset), the patient presented with diarrhea for four days. She reported headaches and double vision were not as severe as before. No thrombus was found in follow-up MRBTI (Fig. 5: e, f). ICP detected by lumbar puncture was 210 mmH_2_O. Immunological tests revealed constant positive anti-β2GPI antibodies (47 RU/mL), and low titers of serum complements C3 and C4. As every onset of high ICP is associated with infection and high titers of immunological items, we presumed that the stenosis might result from autoimmune-mediated inflammation. In a repeated serological test, anti-β2GPI antibodies were still positive, while ANA turned positive for the first time (1: 320). A confirmatory diagnosis of primary APS was made. She then underwent immunomodulatory therapy with prednisone at 5 mg/day and hydroxychloroquine at 5 mg bid. Moreover, Rivaroxaban at 10 mg/day and aspirin 100 mg/day were given to prevent thrombus formation.

Six-month of outpatient neuroimaging follow-up, including CT, MRBTI, CE-MRV, DWI, TCD, and carotid ultrasound), showed no abnormalities in both cerebral arteries and veins (Fig. 5: g, h). Former symptoms of headache and diplopia disappeared completely. The APTT decreased to 48.5 s. Levels of serum complements C3 and C4 remained low. The clinical course was summarized in Fig. 3.

## Discussion

Our previous work firstly classified IJVS based on its etiology, including thrombotic IJVS and non-thrombotic IJVS (bone/vessel/lymph node compression-related IJVS and autoimmune disease-related IJVS) [4]. It is well known that venous thrombosis is the most common complication of APS [5, 6]. To our knowledge, this study reported non-thrombotic IJVS as a complication of APS for the first time. Whereby this report is of great significance to understand the relationship between IJVS and APS.

With regard to the pathogenesis of APS, resent studies indicated that most aPL was directly targeted at phospholipid-binding proteins [2]. Although aPL is a heterogeneous group of antibodies, anti-β2GPI antibodies play a central role in the development of APS. They recognize β2GPI on the surface of endothelial cells and immobilized platelets, then leading to cellular activation and expression of procoagulant activity [7]. Furthermore, the effect of lupus anticoagulants (LA) is also realized through β2GPI or anti-β2GPI antibodies [8, 9]. Anti-β2GPI antibodies are associated with a higher risk of thrombosis than anticardiolipin (aCL) or anti-prothrombin antibodies [8, 9]. Other mechanisms implicate that aPL is also related to interfering with the function of coagulation factors [10–12] and complement-mediated neutrophil activation [13, 14]. In sum, the interaction between aPL and endothelial cells, coagulation cascade (primary and secondary), and inflammatory cells could result in vessel wall damage, contributing to thrombosis or stenosis.

The evaluation of thrombotic risk in patients with APS was usually based on the level of plasma aPL [15]. However, some patients with previously positive aPL profile would turn negative, which then became a challenge for the physicians to evaluate the thrombotic risk. Besides, Bazzan et al. found that despite appropriate anticoagulant treatment, the thrombotic recurrence rate in APS has been reported as high as 7.5/100 patient-years in the first five years after the first thrombotic event [5]. Nevertheless, sometimes there are exceptions due to long-term anticoagulation, such as the case 2 of this study, whose aPL levels were in a persistently moderate tilter (> 40 RU/mL) without recurrence of thrombotic events. Although standardized anticoagulation successfully prevented thrombus formation, the risk of non-thrombotic IJVS, which might result from irreversible immune damages of vascular endothelium caused by aPL, was still existed.

Compared with case 1, case 2 did not receive corticosteroid for APS intervention before referred to our department. Her persistent moderate level of anti-β2GPI antibodies could cause more severe damage to the vessel wall, which might explain her recurrence of symptoms and refractory high ICP.

A combination of anti-Xa inhibitor (Rivaroxaban) and corticosteroid (prednisolone) was prescribed to the two patients. A previous study indicated that endovascular stenting was a promising therapeutic option for focal stenosis of IJV [16]. However, we did not perform stenting/balloon angioplasty in case 1, as the degree of her IJVS was moderate, and the risk of stenosis aggravation was relatively low under the good control of her primary disease.

There is still controversy on the choice of anticoagulation. The vitamin K antagonist (VKA), most commonly warfarin, is widely used for APS-mediated venous thrombus [2]. Whereas, VKA has many limitations in practical use, such as its interaction with food and drug, bleeding complications, and the need for frequent monitoring. Moreover, aPL may affect thromboplastin reagents, tending to affect the international normalized ratio measurement. Rivaroxaban is an effective and safe alternative to warfarin in patients with atrial fibrillation and venous thromboembolism. However, a prospective randomized trial evaluated the efficacy of Rivaroxaban (20 mg/day) in high-risk APS patients (with triple aPL-positive level), which showed that Rivaroxaban was associated with an increased arterial thrombotic risk of events compared with warfarin (www.clinicaltrials.gov; NCT02157272) [17]. A suboptimal drug concentration may account for the thromboembolic complications during this trial. Therefore, we prescribed Rivaroxaban at its relatively low dose (10 mg/day) to test the optimal dose for the patient with negative aPL. Moreover, several studies have shown the benefit of immunosuppressive drugs, especially steroids. Rodriguez et al. discovered that the materno-fetal prognosis could be improved by the addition of low-dose prednisolone during the first trimester of pregnancy in women with APS [18]. Cervera et al. summarized the clinical approach to treat catastrophic APS as a combination of anticoagulation plus steroids plus plasma exchange or intravenous immunoglobulins [6]. Sugie et al. reported that one case of CVST in APS was treated successfully by the combined use of Rivaroxaban and prednisolone [19]. However, to avoid deterioration of IJVS and the occurrence of new-onset venous thrombosis due to steroid-induced hypercoagulability, we administered low-dose prednisone (equivalent to 0.35 mg/kg and 0.55 mg/kg in case 1 and case 2, respectively).

To sum up, these two cases reminded us that firstly, except for thrombotic events, there was non-thrombotic venous stenosis resulted from aPL-mediated vessel wall damage. IJVS may be a rare complication of APS, with clinical features similar to IIH. Secondly, a high titer of aPL could induce vessel wall damage despite long-term standardized anticoagulation for thrombus prevention. Thus, the follow-up of autoantibodies should be done dynamically. Thirdly, concomitant use of anticoagulants and steroids is suggested as the mainstay therapy. Long-term anticoagulation might prevent thrombus formation, and corticosteroid might attenuate aPL-mediated venous wall damage. Last but not least, the MRBTI was a non-invasive and accurate imaging tool on distinguishing intravenous thrombus from non-thrombotic stenosis caused by immune-mediated inflammation. A combination of CE-MRV and MRBTI may be the first option to identify non-thrombotic IJVS precisely and non-invasively, even though DSA is still considered as the golden standard to make a confirmatory diagnosis at present. Whereby, we highly suggest future studies to shed more attention on early diagnosis and treatment in IJVS patients with immunological etiology.

This study revealed that non-thrombotic IJVS was a rather rare complication of APS for the first time. High titer of aPL could induce non-thrombotic IJVS despite use of standardized anticoagulation. Dynamic monitoring of aPL is necessary in APS patients. Combination of anticoagulation and corticosteroids may effectively prevent deterioration of APS-related IJVS.

## Supplementary Information

**Additional file 1 Table S1.** Parameters of jugular ultrasound in **case 1**. **Table 2.** The results of blood and cerebral spinal fluid examination of **Case 2** on admissions.

## Data Availability

The data and materials related to these two cases are available on request to the corresponding author.

## Abbreviations

APS: Antiphospholipid syndrome
IJVS: Internal jugular vein stenosis
aPL: Antiphospholipid antibodies
BMI: Body mass index
ANA: Antinuclear antibodies
ANCA: ntineutrophil cytoplasmic antibodies
TCD: Transcranial Doppler
MRV: Magnetic resonance venography
MRBTI: Magnetic resonance black blood thrombus imaging
DSA: Digital subtraction angiography
ICP: Intracranial pressure
APTT: Activated partial thromboplastin time
LPOP: Lumbar puncture opening pressure
aCL: Anticardiolipin
VKA: Vitamin K antagonist
anti-β2GPI: Anti-β2-glycoprotein-1
IIH: Idiopathic intracranial hypertension
ICP: Intracranial pressure
