# Supplementary material for: The antiphospholipid syndrome may induce non-thrombotic internal jugular vein stenosis: two cases report
Source: BMC Neurol. 2021 Jan 7;21:9. doi: 10.1186/s12883-020-02035-1 (PMC7789703; doi:10.1186/s12883-020-02035-1)
Supplement: Supplementary file 1 — Additional file 1 Table S1. Parameters of jugular ultrasound in case 1. Table 2. The results of blood and cerebral spinal fluid examination of Case 2 on admissions. [file 12883_2020_2035_MOESM1_ESM.docx]

**Supplementary Table 1.** Parameters of jugular ultrasound in **case 1**

| Side | Diameter (mm) | Flow rate (cm/s) | Flow volume (mL/minute) |
| --- | --- | --- | --- |
| Left IJV-J3 segment | 4.3 | 0.86 | 30 |
| Right IJV-J3 segment | 1.7 | 45.94 | 250 |

**Supplementary Table 2.** The results of blood and cerebral spinal fluid examination of **Case 2** on admissions

| **Complete Blood Count** |  |  | **Biochemical examination** |  |  | **Immunity examination** |  |  |
| --- | --- | --- | --- | --- | --- | --- | --- | --- |
| WBC | 5.36 | /μL | T-P | 75.55 | g/L | CRP | 1.4 | mg/L |
| Hb | 137 | g/dL | Alb | 42.30 | g/L | RF | ＜20 | IU/mL |
| PLT | 39 | /μL | Glu | 4.44 | mmol/L | C3 | 0.61 | g/L |
| Neu | 3.05 | /μL | BUN | 2.34 | mmol/L | C4 | 0.11 | g/L |
| Lym | 1.96 | /μL | Cre | 50 | μmol/L | ANA | 1:1000 |  |
|  |  |  | Na | 135 | mmol/L | Anti-ds DNA Ab | Negative |  |
| **Coagulation** |  |  | K | 4.31 | mmol/L | Anti-Sm Ab | Negative |  |
| PT-INR | 1.00 |  | T-Bil | 16.60 | μmol/L | Anti-RNP Ab | Negative |  |
| APTT | 87.0 | sec | AST | 80 | IU/L | ANCA | Negative |  |
| - | 1.10 | μg/mL | ALT | 18 | IU/L | P-ANCA | Negative |  |
| Protein C | 110 | % | LDH | 143 | IU/L | Anti-Cardiolipin A | ＜10 | RU/mL |
| Protein S | 35 | % | γ-GTP | 18 | IU/L | Anti-β2GPI Ab | 50 | RU/mL |
|  |  |  | ESR | 17 | mm/hr |  |  |  |
| **Others** |  |  | T-cho | 3.10 | mmol/L | **Cerebral Spinal Fluid** |  |  |
| Vitamin B12 | 336 | pg/mL | HDL-cho | 1.36 | mmol/L | Appearance | Clear |  |
| Folate acid | 3.9 | ng/mL | LDL-cho | 1.37 | mmol/L | Pressure | 330 | mmH2O |
|  |  |  | TG | 1.34 | mmol/L | Cell | 1*10^6 | /L |
|  |  |  | Lp (a) | 1.36 | mg/dL | Protein | 24 | mg/dL |
|  |  |  | Homocysteine | 13.8 | μmol/L | Glucose | 48.42 | mg/dL |
|  |  |  |  |  |  | Cl | 126 | mEq/L |

**Note:** PT-INR: prothorombin time-International Normalized Ratio; APTT: activated partial thromboplastin time; ANA: anti-neucler antbody; RF: rhematoid factor; Ab: Antibody; RNP: ribonucleoprotein; ANCA: neutrophil cytoplasmic antibody; C3: complement 3; C4: complement 4; Cl: chlorine; AT III: antithorombin III; LP(a): lipoprotein (a)
